# Muscle-Skeletal Abnormalities and Muscle Oxygenation during Isokinetic Strength Exercise in Heart Failure with Preserved Ejection Fraction Phenotype: A Cross-Sectional Study

**DOI:** 10.3390/ijerph19020709

**Published:** 2022-01-09

**Authors:** Amanda Vale-Lira, Natália Turri-Silva, Kenneth Verboven, João Luiz Quagliotti Durigan, Alexandra Corrêa G. B. de Lima, Martim Bottaro, Gaspar R. Chiappa, Dominique Hansen, Gerson Cipriano

**Affiliations:** 1 Rehabilitation Sciences Program, University of Brasília, Brasília 72220-275, Brazil; joaodurigan@gmail.com (J.L.Q.D.); ciprianeft@gmail.com (G.C.J.); 2 Health and Technologies in Health Sciences Program, University of Brasília, Brasília 72220-275, Brazil; natalia.turri@hotmail.com (N.T.-S.); alexandra.lima@gmail.com (A.C.G.B.d.L.); 3 BIOMED-REVAL (Rehabilitation Research Centre), Faculty of Rehabilitation Sciences, Hasselt University, 3590 Hasselt, Belgium; kenneth.verboven@uhasselt.be (K.V.); dominique.hansen@uhasselt.be (D.H.); 4 Faculty of Physical Education, University of Brasília, Brasília 70910-900, Brazil; martim.bottaro@gmail.com; 5 Human Movement and Rehabilitation Graduate Program, Universidade Evangélica de Goiás, Anápolis 76385-608, Brazil; gaspar.chiappa@gmail.com; 6 Heart Centre Hasselt, Jessa Hospital, 3500 Hasselt, Belgium

**Keywords:** microcirculation, muscle strength, spectroscopy, near-infrared, ultrasonography, heart failure

## Abstract

Exercise intolerance, a hallmark of patients with heart failure (HF), is associated with muscle weakness. However, its causative microcirculatory and muscle characteristics among those with preserved or reduced ejection fraction (HFpEF or HFrEF) phenotype is unclear. The musculoskeletal abnormalities that could result in impaired peripheral microcirculation are sarcopenia and muscle strength reduction in HF, implying lowered oxidative capacity and perfusion affect transport and oxygen utilization during exercise, an essential task from the microvascular muscle function. Besides that, skeletal muscle microcirculatory abnormalities have also been associated with exercise intolerance in HF patients who also present skeletal muscle myopathy. This cross-sectional study aimed to compare the muscle microcirculation dynamics via near-infrared spectroscopy (NIRS) response during an isokinetic muscle strength test and ultrasound-derived parameters (echo intensity was rectus femoris muscle, while the muscle thickness parameter was measured on rectus femoris and quadriceps femoris) in heart failure patients with HFpEF and HFrEF phenotypes and different functional severities (Weber Class A, B, and C). Twenty-eight aged-matched patients with HFpEF (*n* = 16) and HFrEF (*n* = 12) were assessed. We found phenotype differences among those with Weber C severity, with HFrEF patients reaching lower oxyhemoglobin (O_2_Hb, μM) (−10.9 ± 3.8 vs. −23.7 ± 5.7, *p* = 0.029) during exercise, while HFpEF reached lower O_2_Hb during the recovery period (−3.0 ± 3.4 vs. 5.9 ± 2.8, *p* = 0.007). HFpEF with Weber Class C also presented a higher echo intensity than HFrEF patients (29.7 ± 8.4 vs. 15.1 ± 6.8, *p* = 0.017) among the ultrasound-derived variables. Our preliminary study revealed more pronounced impairments in local microcirculatory dynamics in HFpEF vs. HFrEF patients during a muscle strength exercise, combined with muscle-skeletal abnormalities detected via ultrasound imaging, which may help explain the commonly observed exercise intolerance in HFpEF patients.

## 1. Introduction

Cardiovascular disease (CVD) is the most important cause of premature mortality, leading to over 17 million deaths yearly around the globe [1,2,3]. Heart failure (HF) is a clinical syndrome characterized by ranks as the second most prevalent CVD [4], exhibiting typical clinical symptoms (i.e., breathlessness, fatigue, ankle swelling) or signs (i.e., pulmonary crackles, peripheral edema, elevated jugular venous pressure) in association with key structural and functional cardiac abnormalities, resulting in a reduced cardiac output and elevated intracardiac pressures at rest or during exercise [5]. Left ventricular ejection fraction (LVEF) assessment using transthoracic echography has been utilized to distinguish patients with preserved or reduced ejection fraction phenotype (HFpEF and HFrEF, respectively). This differentiation is required due to different etiologies, demographics, comorbidities, and therapeutic responses [5]. HFpEF and HFrEF patients typically present similar cardiorespiratory fitness reductions and worse morbidity, hospitalization, and five-year mortality rates compared to healthy individuals [6,7]. Although HFrEF has been widely explored, approximately one-half of all patients exhibit preserved ejection fraction ≥50%, thus reinforcing the need for a better understanding of exercise intolerance in HFpEF patients [8].

Although peripheral mechanisms have been related to exercise tolerance in HF patients [9,10,11], its etiology remains poorly understood in HFpEF [7,12]. As the muscular component has become a key factor in these peripheral mechanisms, it may differ according to the HF phenotype [5,13,14]. The muscular microcirculatory contributions to exercise tolerance amongst different HF phenotypes are poorly understood, particularly when submitted to muscular stress. A better understanding of muscular microcirculatory dynamics is pivotal in unraveling the integrative pathophysiology of exercise intolerance in these patients. 

Skeletal muscle dysfunction is widespread in HF patients. A possible kind of musculoskeletal abnormality that could result in impaired peripheral microcirculation is the loss of muscle mass (sarcopenia) characterized by the atrophy of skeletal muscle, resulting in muscle strength reduction [15,16]. Sarcopenia and muscle strength reduction in HF imply lowered oxidative capacity and perfusion, which affect transport and oxygen utilization during exercise, an essential task of the microvascular muscle function [17,18,19]. Besides that, skeletal muscle microcirculatory abnormalities have also been associated with exercise intolerance in heart failure patients who also present skeletal muscle myopathy [20,21].

Considering the skeletal muscle plasticity and the potential for peripheral adaptation when following exercise-based interventions, changes related to muscle function (such as mass, strength, quality, or microvascular function) may be more noticeable when compared to changes in cardiopulmonary variables [22,23]. Previous research demonstrated that peripheral skeletal muscle dysfunction majorly affects the exercise tolerance in HFrEF patients [10,22,24,25,26]. Poole et al. 2018 [12] demonstrated that there is an important deficit in muscle function in both phenotypes (HFrEF and HFpEF). However, in HFrEF patients, exercise intolerance is related to impaired vascular function, capillary rarefaction, and the absence of red blood cells flux in a considerable proportion of capillaries at rest and during contractions, decreased nitric oxide bioavailability, reduced microvascular oxygen pressures, and elevated muscle deoxygenation. While in HFpEF patients, muscle microvascular dysfunction and oxygenation seem to be more pronounced [24,27] due to expected HF-related peripheral adaptations, such as vascular rarefactions, skeletal muscle abnormalities [28], and higher regional adipose distribution [29].

Of interest, skeletal muscle perfusion reduction in HFpEF patients may worsen oxidative capacity and inflammatory stress, which have been strongly associated with disease-related muscle loss [30,31]. In this sense, assessing the muscle quality [32] and local microcirculatory response differences among HFpEF and HFrEF patients and the association with muscle strength and cardiorespiratory fitness is clinically relevant [33], paving new secondary prevention and rehabilitation treatment alternatives [34]. A recent study described higher quadriceps echo intensity, muscle thickness (cm), and muscle mass (Kg) values in HF patients compared to a control group and its association with poor exercise capacity in HF [35]. The EI of the quadriceps femoris correlated physical performance in sedentary older adults and was the strongest predictor of this functional test, with 30% of the variance explained by the EI [36,37,38].

This study primarily aims to explore the mechanisms leading to exercise intolerance by comparing the local oxygen extraction response during muscle strength exercise and ultrasound-derived parameters among different HF phenotypes with different disease severities. Secondarily, it intends to analyze possible associations between exercise-induced tissue saturation index response and ultrasound-derived variables with (a) an isokinetic muscle strength test and (b) cardiorespiratory fitness. The outcome measures will be explored according to HF phenotypes and disease severities.

We hypothesized that peripheral dysfunctions are more pronounced in patients with HFpEF compared to HFrEF because of a lower oxygen extraction capacity and poor muscle quality [24,27]. 

## 2. Materials and Methods

### 2.1. Study Design and Participants

This is a cross-sectional observational study that followed the STROBE recommendations [39]. The advisors were blinded for the different subgroups only for sample characterization variables. The study was performed in accordance with the Declaration of Helsinki (2013) (approved by the ethical committee of the University of Brasília, CAAE 81309417.7.0000.8093). After a careful explanation of the nature and risks of the experimental procedures, all participating patients provided informed consent before starting the measurements. The study was realized between June 2018 and September 2019 at the University of Brasília.

Male and female individuals from a convenient sample, diagnosed with HFpEF or HFrEF, stable and under optimal medical treatment, were recruited and allocated by phenotype. The inclusion criteria were: (1) minimal age of 35 years; (2) at least six months of HFrEF or HFpEF diagnosis [5]; (3) HF with ischemic, hypertensive, or idiopathic etiology; (4) clinically stable for at least three months; and (5) a sedentary lifestyle (in the last six months). The exclusion criteria were: (1) clinically diagnosed pulmonary, inflammatory, musculoskeletal, or orthopedic diseases precluding exercise performance; and (2) functional New York Heart Association (NYHA) [40] class IV. 

All participants were assessed during four experimental visits. The first visit was directed to clinical assessment, body composition, and pulmonary function; the second to echocardiogram assessment; and the third for muscle ultrasound and cardiopulmonary exercise testing. Finally, a fourth visit was planned to assess the isokinetic muscle strength and local oxygen with near-infrared spectroscopy (NIRS).

### 2.2. Baseline Clinical Characteristics

Patients were evaluated by a cardiologist who collected detailed information about the clinical history, diagnosis, and current symptoms. The NYHA [40] and Weber [41] functional classification was included to provide complementary clinical information regarding HF severity. The whole-body composition was assessed using dual-energy X-ray absorptiometry (DXA), cardiac function using echocardiography, pulmonary function via spirometry, and cardiorespiratory fitness via cardiopulmonary exercise test (CPX). 

#### 2.2.1. Dual-Energy X-ray Absorptiometry (DXA) 

The whole-body composition was estimated by using DXA (Lunar Prodigy Bone Densitometers, GE Healthcare, Chicago (Illinois), United States), with a full-body examination. Fat and lean mass were expressed in absolute values (kg), and percentage values (%) described by the DXA scan manufactured. The participants were not instructed on food intake or nutritional prescription.

#### 2.2.2. Echocardiography

The echocardiographic measurements were performed according to the recommendations of the American Society of Echocardiography [42]. Classic parameters representing cardiac structure (left atrial enlargement and/or left ventricular hypertrophy) and function (ejection fraction calculation followed Simpson method) were evaluated [5]. All patients were evaluated by the same cardiologist using an ultrasound (Vivid S60, GE Healthcare, Tirat Carmel (Haifa), Israel) and probe (matricial 4V; setorial 3Sc) with both GE 3Sc-RS Probe (Sector) and the GE 4V-D Probe (collector). Resting supine position included the following measurements: left ventricular ejection fraction (LVEF, %), left atrial volume index (LAVI, mL/m^2^); left ventricular mass index (LVMI, g/m^2^); pulsed wave tissue Doppler imaging was used for early diastolic velocity (e’) at the septal annulus. The E/e ratio was measured as an indicator for LV filling pressures.

#### 2.2.3. Spirometry

Lung volumes and capacities were assessed by forced spirometry, using s calibrated spirometer (MicroLab CareFusion^®^ MK8 ML 3500; CareFusion, Yorba Linda, United States) through a proper Spirometry PC Software, version 2.2 (Williams Medical Supplies Ltd., Rhymney (South Wales), UK). The spirometry evaluations followed the American Thoracic Society/European Respiratory Society’s recommendations, 2005 [43]. The predictions were calculated according to the equations for the Brazilian population according to Pereira et al., 2007 [44]. Variables considered were forced expiratory volume in the first second (FVE_1_, L/s), forced vital capacity (FVC, L/s), ratio FVE_1_ by FVC (FVE_1_/FVC, L/s), and all predicted value (%).

#### 2.2.4. Cardiopulmonary Exercise Test

Functional exercise capacity was assessed utilizing a maximal incremental cardiopulmonary exercise test (CPX) of an electromagnetic bicycle (Corival, LODE BV Medical Technology, Groningen, The Netherlands) under cardiologist supervision. The gas analyzer (CPET, Cosmed, Rome, Italy) followed the breath-by-breath method, evaluating the variables determined by both V-slope and ventilatory equivalents method [45], thereby assessing peak oxygen uptake capacity (peak VO_2_) and peak power using a 1-minute work stage protocol (starting workload of 20W and incremental workload of 10 to 15 W). Oxygen uptake and heart rate (12-lead electrocardiogram) measurements were performed continuously. All patients cycled until volitional exhaustion, when patients were no longer able to maintain a cycling frequency of 55 rpm higher. Peak exercise effort was confirmed when respiratory gas exchange ratio (RER) was ≥1.10, with dyspnea or leg or general fatigue.

The exercise test occurred at least 2–3 h following the last meal, and the patients could not exercise 24 h before the test. Primarily, patients adopted a rest period on the ergometer of at least 5 min, until a steady-state respiratory had been established. At the end of the exercise, the state of recovery was observed for 2 min. All individuals performed the exercise test on a symptom-limited ramp by increasing the standard ramp’s work rate. After a warm-up period of 2 min at 20 W, an increase in the work rate at a slope of 10–15 W/min was stated (recommendation for HF patients) [46]. Individuals were asked about their perception of ventilatory effort and muscular fatigue every 2 min, according to the Borg scale (6 to 20) [47]. The VE/VCO_2_ was expressed as a slope value, calculated by the linear regression (y = mx + b, b = slope) of the exercise curve from the beginning to the peak of the effort according to Arena, R. et al. 2004 [48]. The determination of RER above 1.1 is defined as a test quality criterion, confirming that the individual has reached the maximum effort. The predicted VO_2_ max was determined by the equation of Jones and Campbell, 1982 [49,50], as follows for males: predicted VO_2_ max = [60.0 − (0.55 × age)] × 1.11; and for females: predicted VO_2_ max = [48.0 − (0.37 × age)] × 1.11.

### 2.3. Isokinetic Muscle Strength Test

Isokinetic muscle strength tests were performed using the Biodex system III Isokinetic Dynamometer (Biodex Medical, Inc., Shirley, NY, USA). The dynamometer arm’s rotation axis was adjusted to the right knee, and velcro belts were used to secure the thigh, pelvis, and trunk to the chair to prevent compensatory body movement. The lateral femoral epicondyle was used as the bony landmark for matching the knee joint with the axis of rotation of the dynamometer resistance adapter. Gravity correction was obtained by measuring the torque exerted on the dynamometer resistance adapter with the knee in a relaxed state at full extension. Patients were instructed to fully extend and flex the knee and work maximally during each exercise set. Verbal encouragement was given throughout the test session.

Isokinetic muscle strength assessment protocol comprised 20 repetitions, requiring maximum concentric effort at an angular velocity of 180°/s. Patients performed six initial submaximal repetitions for familiarization purposes. After three minutes of rest, the isokinetic muscle strength test was performed [18,19,51,52,53,54]. Variables analyzed were peak torque (Nm) and adjusted per body weight ratio (Nm.kg), total repetition maximum work (J) and adjusted per body weight ratio (%), total work (J), work fatigue (%), and average power (W).

### 2.4. Near-Infrared Spectroscopy (NIRS)

During isokinetic muscle strength testing, a near-infrared spectroscopy (NIRS) device with a dual-wavelength (760 and 850 nm), continuous-wave system type, containing three pairs of LEDs configured for spatially resolved spectroscopy (SRS) with a source–detector spacing of 30, 35, and 40 mm were utilized to assess local oxygen extraction response (Portamon for OxySoft 3.0.95, Artinis Medical Systems, Amsterdam, The Netherlands). Changes in absorbance were recorded using the oxyhemoglobin (O_2_Hb, μM) and deoxyhemoglobin (HHb, μM) values to assess the oxygenation status of the muscle [55]. In addition, the tissue saturation index (TSI, %) was calculated from the absorption of coefficients derived from the attenuation of light at different source–detector distances and wavelengths as a relative value (%), which is feasible for comparing and evaluating the achievement of critical limits during exercise. For this, the equipment was positioned on the right leg vastus lateralis (approximately 5 cm from the lateral patellar border) and covered with a dark blue elastic band to avoid interference from ambient light and adhesive tape without pressing the equipment. The data were sampled at 10Hz and stored for offline analysis using the LabChart Pro v8 software (ADInstruments, Sidney, Australia).

Data were extracted from the NIRS software in excel (the data were sampled at 10 Hz). Afterward, and according to the timestamps manually performed during the assessment, we extracted the necessary information for data analysis (for example, the data referring to the time used to position the patient or check the signal was excluded). After this process, the data were transferred to the LabChart Pro v8 software (ADInstruments, Sydney, Australia). This software assisted us in graphically revising the extracted NIRS data. We carried out this process because by just looking up values in the spreadsheet, it would not be possible to visualize the continuous waves of the evaluated variables. Thus, we were able to relate the timing during assessment and the behavior of the constant waves. In this way, we determined the stretches of time in each phase of the test that would be considered for the statistical analysis. 

For the interpretation of NIRS data, it is important to remember the behavior of the variables during exercise. The TSI continuous wave drops during exertion and returns to its baseline condition after exertion. The O_2_Hb continuous wave behaves similarly to the TSI. The HHb continuous wave is different from the previous ones, as it increases during exertion and falls after exertion, returning to its basal condition or close to it.

For statistical analysis and graph signal processing analysis of the NIRS curve, baseline (mean obtained value for the 30s of the resting phase), exercise (lowest obtained value for TSI, %, and O_2_Hb, μM and highest for the HHb, μM) with a maximum interval variation acceptance of 4 s (20 to 24 s, depending on manual NIRS mark) and recovery (highest obtained value for TSI, % and O_2_Hb, μM and lowest for the HHb, μM) were considered as time points for comparison [30,56]. An individual visual inspection of the curves was made to exclude possible failures or noise from the graph signal. Then, eligible individuals were analyzed and presented on graphs that included the individual mean values from each variable (representative cases). Heart rate (HR, bpm), systolic (SBP, mmHg), and diastolic blood pressure (DBP, mmHg) were also monitored before and after the isokinetic muscle strength test to assess hemodynamic parameters.

### 2.5. Ultrasound-Derived Measures: Echo Intensity and Muscle Thickness

The ultrasound images were captured by using an ultrasound device (HD11XE, Phillips, Amsterdam, The Netherlands) with a 7.5 MHz linear matrix transducer. The individuals were evaluated in a supine position with the knee in passive flexion with a 15-centimeter under-knee support and neutral rotation. The images were always acquired on the right leg with the transducer placed transverse and perpendicular to the long axis of the anterior thigh, rectus femoris (RF), and vastus lateralis (VL) muscles (50% of the distance between the iliac spine anterior superior to the superior edge of the patella) to assess muscle thickness, using appropriate transmission gel [57]. The ultrasound was consistent in every examination since the parameter was set at 60 mm of depth, with a preset of gain of 38 Gn, dynamic range of 232 dB, and pulse repetition frequency of 21 Hz.

The images were analyzed using the ImageJ software (1.52q version, Bethesda, EUA) [58]. The quadriceps femoris was analyzed between the uppermost part of the femur and the superficial fascia of the rectus femoris (which includes the rectus femoris and vastus intermedius) and the isolated rectus femoris [59,60]. The measurement of echo intensity was determined by a grayscale analysis using ImageJ software. The region of interest was selected for each assessed muscle, including all muscle areas and removing bone or surrounding fascia from the selected area [59]. The mean of grayscale was calculated using an 8-bit resolution measure, resulting in a number between 0 = black and 255 = white. An average of the three measurements per muscle was calculated. In the quadriceps femoris, only the rectus femoris muscle was used for analysis [59,60]. Patients were instructed not to perform any physical activities 24 h before testing.

### 2.6. Statistical Analysis 

Data are expressed as mean ± standard deviation (SD), absolute (n), or relative frequencies (%). Shapiro–Wilk test was used to indicate sample data distribution. Parametric or non-parametric tests were applied accordingly. Group differences for continuous outcome variables were compared using unpaired t (mean difference and 95% confidence interval) or Mann–Whitney U test (Hodges-Lehmann’s difference). Categoric variables were compared using Fisher’s exact test. 

We performed a bivariate correlation (Spearman’s or Pearson’s) analysis to investigate the associations between exercise-induced tissue saturation index response (TSI, %) and ultrasound variables (echo intensity (EI, 0–255) and muscle thickness MT, cm) of rectus femoris (RF), with isokinetic muscle strength (PT, Nm) and cardiorespiratory fitness (peak VO_2_, mL^−1^.min^−1^) among HF phenotypes (HFpEF and HFrEF) and severity of functional impairment classification (Weber A + B and Weber C). Association levels were defined according to correlation coefficient (r) (0.00 no association; 0.20 weakly; 0.50 moderately; 0.8 strongly and 1.00 perfectly) [61] or (rho) (0.00 to 0.20 negligible; 021 to 0.40 weak; 0.41 to 0.60 moderate; 0.61 to 0.80 strong and 0.81 to 1.00 very strong) [62].

As a preliminary study, and considering the absence of similar studies involving microcirculatory dynamics within resistance exercise in HF, we included the post hoc analysis to detect the power calculation of the study (effect size) and present in the results. The effect size and power for groups comparisons were estimated using G*Power Software 3.1. These parameters were chosen because their statistical difference was significant (*p* < 0.05–alpha error).

Statistical software GraphPad Prism (8.4.0, San Diego, CA, USA) was used for statistical analyses and figure production. All analyses considered 95% confidence interval (CI), and statistical significance was set at *p*-value ≤ 0.05 (two-tailed).

## 3. Results

### 3.1. Baseline Clinical Characteristics

Participants’ characteristics are shown in Table 1. Both groups were similar by design regarding age and BMI when comparing both phenotypes by Weber class (*p* > 0.05). Fat mass and lean tissue distribution were similar between HF phenotypes groups and severities subgroups (*p* > 0.05) (Table 1). Meanwhile, in HFpEF patients, Weber Class C presented higher fat body mass and fat leg mass than in HFpEF patients with Weber Class A + B (*p* < 0.05) (Table 1). Comparing the total sample between HFpEF and HFrEF, there were no differences (*p* > 0.05) between risk factors and CVDs in the phenotypes (Table 1). However, HFpEF patients used fewer diuretics compared to HFrEF patients (*p* = 0.020) (Table 1). 

As expected, differences were detected for all echocardiographic parameters between HF phenotypes (*p* < 0.05) (Table 2). Regarding the pulmonary function variables (Table 2), when comparing both phenotypes (HFpEF and HFrEF), Weber Class C presented a lower predicted value of % predicted FEV_1_ (*p* = 0.024) and FEV_1_/FVC ratio (*p* = 0.020) for HFpEF than the HFrEF group. In HFpEF patients, there was a difference in FEV_1_ (L/s), % predicted FEV_1_, and FCV (L) parameters, indicating higher values in the Weber Class A + B than Class C group (*p* < 0.05). Finally, when comparing both HF phenotypes without considering severities, the HFpEF group presented lower values of % predicted FVC and FEV1/FVC ratio (*p* < 0.05).

Regarding the cardiorespiratory fitness (Table 2), subjects presented similar peak VO_2_ (mL·kg^−1^·min^−1^) and VE/VCO_2_ slope (*p* > 0.05), independently of phenotype or disease severity. However, HFpEF presented a higher peak power output (W), predicted peak VO_2_ (%), and peak VO_2_ (mL·min^−1^) than the HFrEF group (*p* = 0.024; *p* = 0.046; *p* = 0.020, respectively). HFpEF with Weber Class A + B patients presented a higher absolute peak power output, peak VO_2_ (mL·kg^−1^·min^−1^), and peak VO_2_ (mL·min^−1^) as opposed to HFrEF with Weber Class A + B (*p* = 0.024; *p* = 0.060; *p* = 0.024, respectively). In the HFpEF analysis, there was a difference among exercise (min), peak power output (W), peak VO_2_ (mL·kg^−1^·min^−1^), and peak VO_2_ (mL·min^−1^) parameters, indicating higher values in the Weber Class A + B than in the Class C group (*p* = 0.001; *p* = 0.0002; *p* < 0.0001; *p* < 0.0001, respectively), while in the HFrEF analysis, there were observed differences in exercise (min), peak power output (W), peak VO_2_ (mL·kg^−1^·min^−1^), predicted peak VO_2_ (mL·Kg^−1^·min^−1^), and peak VO_2_ (mL·min^−1^) variables, indicating higher values in the Weber Class A + B than in the Class C group (*p* = 0.048; *p* = 0.041; *p* = 0.001; *p* = 0.048; *p* = 0.030, respectively).

### 3.2. Peripheral Muscle Microcirculation Dynamics during Isokinetic Muscle Strength Testing 

Twenty-eight patients were analyzed in the study. However, 17 patients were considered for peripheral muscle microcirculation dynamics analysis during isokinetic muscle strength testing due to NIRS device and signal analysis limitations. Baseline tissue saturation index (TSI, %), oxyhemoglobin (O_2_Hb, μM), and deoxyhemoglobin (HHb, μM) were similar between HF phenotypes (HFrEF and HFpEF) and different among severity classifications (Weber Class A + B vs. C) (Table 3; *p* > 0.05). 

During the exercise, the TSI values were not different between HFpEF and HFrEF groups (*p* > 0.05) (Table 3). However, when we consider Weber Class A + B, TSI (%) values tended to be lower in HFrEF patients (44.2 ± 8.2 vs. 36.0 ± 2.4, *p* = 0.060) (Table 3). A trend of lower TSI was found in the HFrEF group when compared to the HFpEF group (44.8 ± 6.6 vs. 57.6 ± 13.7, *p* = 0.161). Within HFrEF with Weber Class C patients, there was a trend of higher TSI value than that in Weber Class A + B patients (44.8 ± 6.6 vs. 36.0 ± 2.4, *p* = 0.071). HFpEF values between Weber Class A + B and C were not different (*p* > 0.05).

During the exercise, the O_2_Hb values were not different between HFpEF and HFrEF groups (*p* > 0.05) or in between phenotypes with Weber Class A + B (>0.05) (Table 3). Among those with Weber C severity, while HFrEF patients reached lower oxyhemoglobin (O_2_Hb, μM) (−10.9 ± 3.8 vs. −23.7 ± 5.7, *p* = 0.029; effect size= 2.6; power = 0.8) during exercise, HFpEF patients maintained lower O_2_Hb during the recovery period (−3.0 ± 3.4 vs. 5.9 ± 2.8, *p* = 0.007; effect size = 2.9; power = 0.9) (Table 3). Altogether, in terms of HFpEF, Weber Class C patients presented (more negative value) a poor capability to reach greater oxygen extraction (oxyhemoglobin, O_2_Hb, μM) during exercise than Class A + B patients (−10.9 ± 3.8 vs. −27.2 ± 9.2; *p* = 0.006). HFrEF values between Weber Class A + B and C were not different (*p* > 0.05).

During the exercise, the HHb values were not different between HFpEF and HFrEF groups (*p* > 0.05)or in Weber Classes A + B and C groups (*p* > 0.05) (Table 3). Moreover, there was a trend towards a higher value of deoxyhemoglobin (HHb, μM) during the exercise phase in HFpEF with Weber Class A + B patients than those with Weber Class C (14.0 ± 6.4 vs. 3.4 ± 7.6; *p* = 0.062). HFrEF values between Weber Class A + B and C were not different (*p* > 0.05).

At the recovery phase, there was no difference found for TSI among phenotypes and subgroups analysis (*p* > 0.05). At the recovery phase, the only statistical difference was found for the comparison among phenotypes indicating a lower O_2_Hb value in the HFpEF Weber Class C group than the HFrEF groups (−3.0 ± 3.4 vs. 5.9 ± 2.8; *p* = 0.007). The HHb values were not different during the recovery phase between HFpEF and HFrEF groups (*p* > 0.05). When comparing both phenotypes within Weber Class A + B, significant differences for HHb (μM) parameter were observed during the recovery phase. Higher values were observed in the HFrEF group compared to HFpEF group (+18.8 ± 4.8 vs. +8.9 ± 5.6, *p* = 0.042; effect size = 1.9; power = 0.6). When comparing both phenotypes with Weber Class C (*p* > 0.05), no difference was found. HFpEF values between Weber Class A + B and C were not different (*p* > 0.05). Within HFrEF, Weber Class A + B patients presented a higher deoxyhemoglobin (HHb, μM) value during recovery than Weber Class C patients (18.8 ± 4.8 vs. 0.7 ± 1.7; *p* = 0.016). 

**Figure 1:**
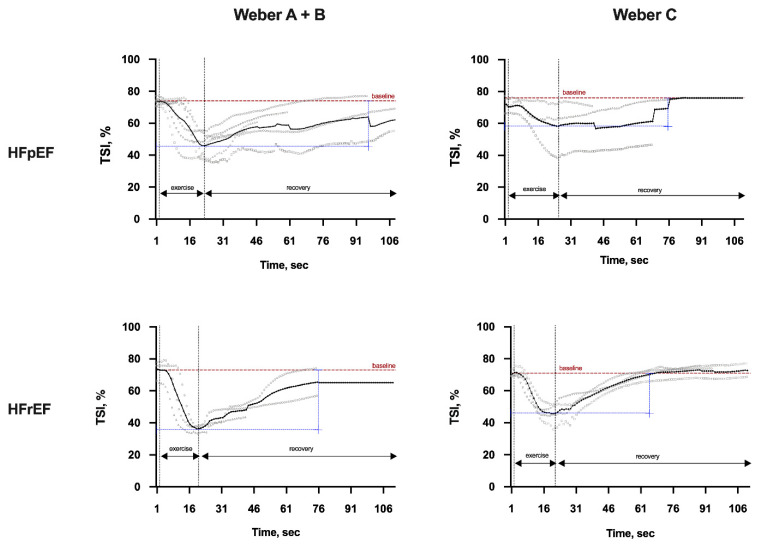
Representative cases of local oxygen extraction (tissue saturation index—TSI, %) during isokinetic muscle strength evaluation by the Weber Class in both heart failure phenotypes. Legend: Average and individual behavior of local oxygen extraction (tissue saturation index) during isokinetic muscle strength maneuver by the Weber Class A + B or C in between heart failure patients’ groups. HFpEF, heart failure with preserved ejection fraction; HFrEF, heart failure with reduced ejection fraction; TSI, tissue saturation index; sec, seconds.

The TSI (Figure 1) recovery period was longer for the HFpEF group compared to the HFrEF group in both Weber A + B and Weber C severity subgroups. Similarly, the recovery period for TSI was significantly longer in Weber A + B patients than in C patients with both HFpEF and HFrEF phenotypes (Figure 1).

**Figure 2:**
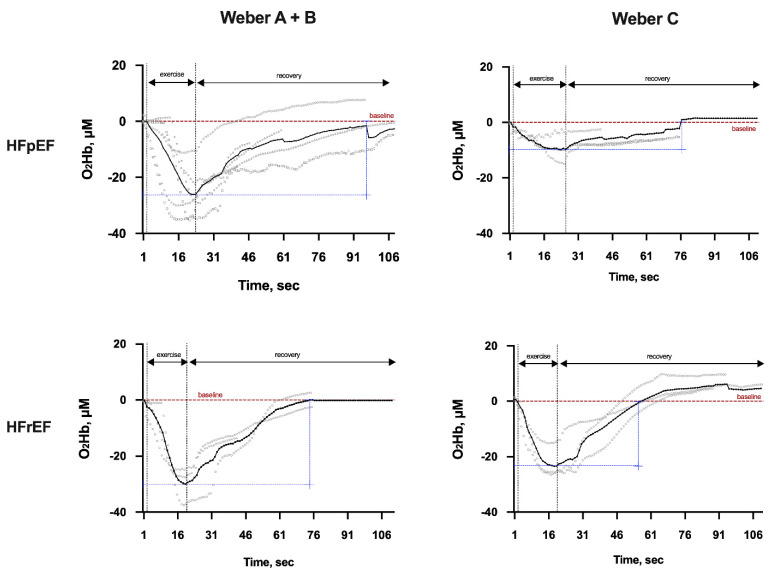
Representative cases of local oxygen extraction (oxygenation-O_2_Hb, μM) during isokinetic muscle strength evaluation by the Weber Class in both heart failure phenotypes. Legend: Average and individual behavior of local oxygen extraction (oxygenation) during isokinetic muscle strength maneuver by the Weber Class A + B or C in between heart failure patients’ groups. HFpEF, heart failure with preserved ejection fraction; HFrEF, heart failure with reduced ejection fraction; O_2_Hb, oxyhemoglobin; sec, seconds; μM, micrometer.

Although the decrease in O_2_Hb during exercise was similar in both phenotypes when considering the severity A + B, the return to baseline was faster in HFrEF patients than in HFpEF patients (Figure 2).

**Figure 3:**
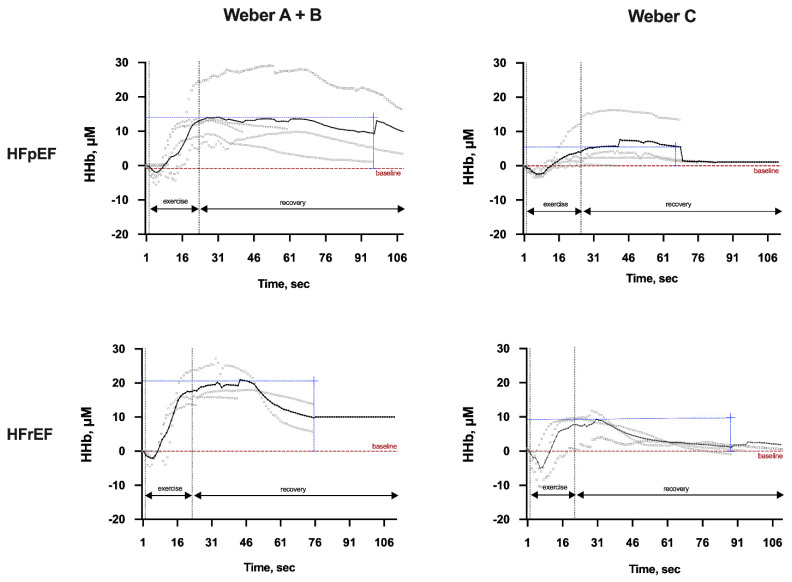
Representative cases of local oxygen extraction (deoxygenation-HHb, μM) during isokinetic muscle strength evaluation by the Weber Class in both heart failure phenotypes. Legend: Average and individual behavior of local oxygen extraction (deoxygenation) during isokinetic muscle strength maneuver by the Weber Class A + B or C in between heart failure patients’ groups. HFpEF, heart failure with preserved ejection fraction; HFrEF, heart failure with reduced ejection fraction; HHb, deoxyhemoglobin; sec, seconds; μM, micrometer.

This faster return to baseline starting in HFrEF patients also occurred when comparing both phenotypes with Weber C severity. In addition, when comparing the severities A + B versus C in HFpEF patients, a greater reduction occurred in Weber Class A + B, although a poor recovery was identified in Class C severity. Among those with Weber Class A + B, HFpEF patients required a longer HHb recovery period after exercise than those with HFrEF (Figure 3). Similarly, in the first seconds of recovery for severity C, HFpEF patients maintained more HHb, while HFrEF patients reduced their values faster. Lastly, HFpEF patients had a worse recovery compared to those with HFrEF, regardless of severity.

**Figure 4:**
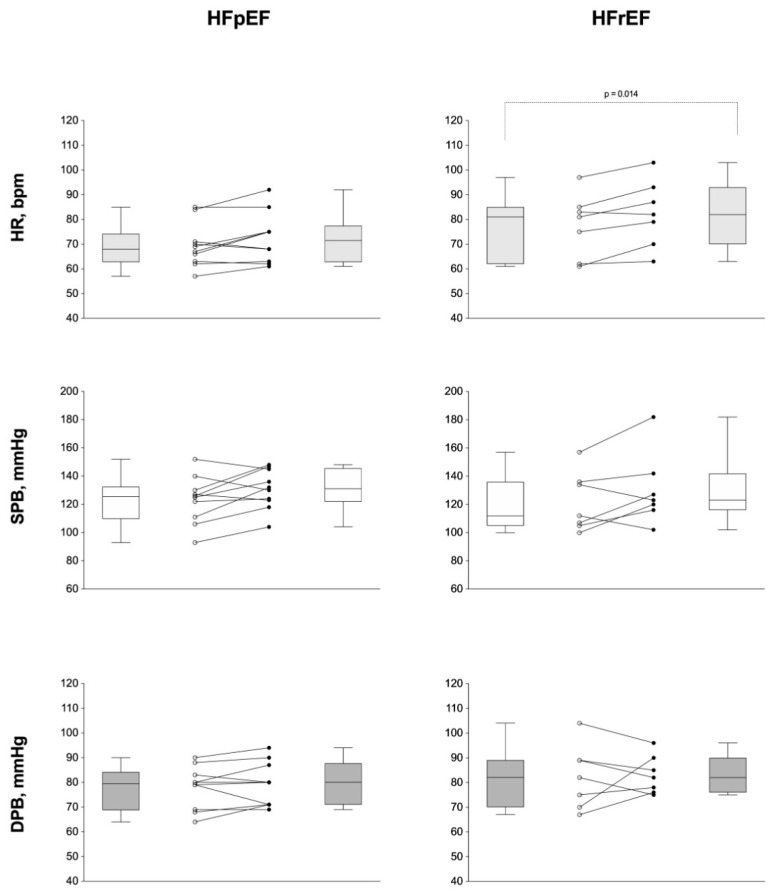
Hemodynamic parameters before and after the isokinetic muscle strength test in both heart failure phenotypes. Legend: Individual behavior of hemodynamic parameters before and after the isokinetic muscle strength test between heart failure groups. HFpEF, heart failure with preserved ejection fraction; HFrEF, heart failure with reduced ejection fraction; HR, heart rate (bpm); SPB, systolic blood pressure (mmHg); DPB, diastolic blood pressure (mmHg).

Figure 4 presents the hemodynamic parameters before and after the isokinetic muscle strength test (systolic blood pressure, SPB; diastolic blood pressure, DPB; and heart rate, HR) and compared them HFpEF and HFrEF groups. There was a statistically significant difference only for the HR parameter in the HFrEF group (*p* = 0.014).

### 3.3. Isokinetic Muscle Strength Parameters 

No isokinetic muscle strength parameters (peak torque, peak torque/body mass, maximal repetition total work, work/body weight, total work, work fatigue, and average power) were different among HF phenotypes or between disease severity states (*p* > 0.05) (Table 4). However, within HFpEF, peak torque (*p* = 0.019), peak torque/body mass (*p* = 0.005), maximal repetition total work (*p* = 0.003), work/body weight (*p* = 0.007), total work (*p* = 0.004), and average power (*p* = 0.019) presented higher values in parameters in Weber Class A + B patients than Weber Class C patients. 

### 3.4. Ultrasound-Derived Parameters (Echo Intensity and Muscle Thickness) 

There was no difference in echo intensity of the rectus femoris between HF phenotypes (*p* > 0.05) (Table 5). However, there was a greater echo intensity value in the HFpEF group with Weber C than the HFrEF group (29.7 ± 8.4 vs. 15.1 ± 6.8, *p* = 0.017). Moreover, HFpEF patients with Weber Class C had a higher echo intensity value than those in Weber Class A + B (14.1 ± 8.7 vs. 29.7 ± 8.4, *p* = 0.009).

Muscle thickness (MT, cm) (Table 5) did not exhibit significant differences between HFpEF and HFrEF groups independent of severities for rectus femoris analysis (*p* > 0.05). In addition, a smaller rectus femoris MT was observed in HFpEF patients with Weber Class C than those with Weber Class A + B (*p* = 0.023).

### 3.5. Associations 

Regarding the association between exercise-induced tissue saturation index response (NIRS) during strength isokinetic testing (TSI, %) and isokinetic muscle strength parameters (PT), there was no correlation for the HFrEF group (Table 6), while a moderate negative correlation was found in the HFpEF group (r = −0.697; *p* = 0.031). Conversely, TSI only correlated with peak VO_2_ (mL·min^−1^) in HFrEF with Weber Class A + B patients (r = 0.999; *p* = 0.010). No other correlations between TSI and Weber Class C or TSI only comparing phenotypes regardless of disease severity were observed.

The associations between the ultrasound-derived measures of RF (echo intensity, EI, 0–255; muscle thickness, MT, cm) with isokinetic muscle strength (PT) and cardiorespiratory fitness (peak VO_2,_ mL·min^−1^) are presented in Table 6. Although a positive correlation was expected between muscle thickness and peak torque, this was not observed in either phenotype. A positive association was only found for the HFrEF group.

RF_EI was moderately negatively associated with PT (r = −0.570; *p* = 0.021) and peak VO_2_ (r = −0.581; *p* = 0.015) in HFpEF patients, but not in the HFrEF phenotype (*p* > 0.005). Associations among RF_EI and isokinetic PT or peak VO_2_ were not found in HF subgroups according to Weber Class (*p* > 0.05) (Table 6). 

A moderate association between RF_MT and isokinetic PT was observed in the HFrEF phenotype (r = 0.778; *p* = 0.03) and Weber Class C subgroup (r = −0.880; *p* = 0.049). Lastly, a moderate association was found between the RF_MT and the peak VO_2_ in both phenotypes considering the entire group (HFpEF: r = 0.672; *p* = 0.004; HFrEF: r = 0.751; *p* = 0.005), which was also observed in the severities subgroups (Weber Class A + B and C), among those with HFpEF (Weber Class A + B: r = 0.687; *p* = 0.020; Weber Class C: r = 0.937; *p* = 0.019) (Table 6).

## 4. Discussion

Our study found poor peripheral oxygen extraction, particularly in HFpEF patients during isokinetic muscle strength testing, which was more pronounced in Weber C patients. Additionally, the HFrEF with Weber Class A + B group presented a worse recovery than the HFpEF group for the HHb parameter. Despite a similar O_2_ extraction during exercise, higher deoxygenation was found during the recovery period. The groups were similar in terms of muscle strength. However, a higher echo intensity value was found only in the HFpEF with Weber Class C group. We believe that intramuscular fat is an important factor to consider when interpreting this result, which corroborates the higher echo intensity value in the HFpEF group. Only the HFrEF with Weber Class C and HFrEF groups had correlated muscle strength and muscle thickness. This could be due to the lower echo intensity value. Moreover, although lower limb muscle strength and ultrasound-derived thickness were not different between subgroups, echo intensity revealed a higher value in HFpEF patients. The fat body mass and legs fat mass were higher in HFpEF with Weber Class C patients. Additionally, echo intensity was negatively associated with cardiorespiratory fitness in the same phenotype. Hence, our preliminary findings suggest that peripheral muscle microcirculation dynamics can affect a strength-type exercise similar to an aerobic-type exercise in HFpEF.

A similar local oxygen response decrease in HFpEF was also observed during the cardiopulmonary exercise test (CPX), highlighting the significant role of impaired arteriovenous O_2_ difference augmentation in contributing to exercise intolerance in the HFpEF population [24]. Moreover, the arteriovenous O_2_ difference is also reduced in HFpEF when performing a hand dynamometer test [63]. Another study evaluated the oxygen response during plantar flexion exercise by magnetic resonance with spectroscopy in HFpEF patients and healthy individuals, revealing a poor performance in HFpEF patients, indicated by a faster decrease in phosphocreatine and consequent impairment in the ATP flow [64,65], possibly causing microvascular damage [66]. In both HF phenotypes, there is a change in the distribution of muscle fiber type, making them more glycolytic than oxidative. However, the reduced arterial–venous O_2_ difference in HFpEF patients may be related to deficiencies in skeletal muscle oxidative metabolism or alteration in microvascular O_2_ transport, generating greater problems of O_2_ extraction, suggesting that this condition is more impaired in HFpEF [67]. Among those with HFpEF, we also confirmed that Weber Class C patients presented a reduced capability to reach a greater oxygen extraction during an isokinetic muscle strength test compared to Classes A and B. Moreover, a longer recovery period was found in HFpEF in both severity classes. A previous study comparing HFpEF and healthy controls identified that the major mechanism underlying the functional impairment in such groups appears to be related to deranged peripheral hemodynamics, including a reduced leg blood flow and vascular conductance [68]. Moreover, higher deoxyhemoglobin values were observed during the recovery phase when comparing both phenotypes within Weber Class A + B. Considering that both strength and HHb (μM) during exercise were similar, the HHb increase in the HFpEF phenotype during the recovery phase may be related to poor peripheral oxygen extraction. There was no desaturation during the tests, despite the differences found in pulmonary function. There was also no need for supplemental O_2_.

The current study thus adds to previous research that HFpEF patients experience significant skeletal muscle oxygen extraction abnormalities during strength-type exercises, contributing to exercise intolerance, particularly when greater (skeletal muscle) exercise intensities are elicited. In relation to hemodynamic parameters, the systolic blood pressure was slightly higher in the HFpEF group and may be related to less damage to cardiac output, as the ejection fraction is normal. In the heart rate of patients with HFrEF is higher, probably because they experience inotropic incompetence due to reduced ejection fraction; thus, they should use the chronotropic response, which is HR elevation.

Higher echo intensity values have been related to an increased intramuscular adipose and connective tissue distribution [69,70,71]. Previous studies have shown that HFpEF presents 30% higher fat tissue and intramuscular fat tissue than the healthy population [28,72,73]. Our study arouses new findings regarding muscular quality differences between HFpEF and HFrEF. We found an increasing trend of the RF echo intensity in the HFpEF phenotype with Weber Class C, and most interestingly, its higher echo intensity was associated with a lower peak VO_2_ in HFpEF participants. Additionally, we found higher body and leg fat mass in HFpEF with Weber Class C patients than those with Weber Class A + B. These findings could demonstrate different mechanisms related to exercise intolerance in HFpEF, suggesting a closer association between muscle quality, disease severity, and exercise intolerance. Furthermore, Nakano et al. [35] described a positive correlation between quadriceps femoris echo intensity and NYHA class and age. A negative correlation between quadriceps femoris echo intensity and peak VO_2_ was also described in HF and healthy subjects [35]. Concerning muscle thickness, a positive association with peak torque was only found in HFrEF participants, reinforcing that HFpEF is peripherally more affected.

A greater quantity of type II muscle fiber in HF and the lower capillarity ratio per fiber had already been related to greater exercise intolerance in previous studies [14,28]. Similarly, a lower amount of type I fiber has been shown to cause a lower peak VO_2_ [14,28]. Furthermore, the reduced oxidative and diffusive capacity combined with a low exercise tolerance in HF patients compared to healthy volunteers suggests that skeletal muscle metabolism is a potentially important target for future HF treatment strategies [65,74,75], providing more assertive and individualized treatment strategies. In this way, oxidative and structural muscle impairment is a possible underlying exercise intolerance mechanism that appears able to impact strength modality in HFpEF patients.

This study contains limitations that might be addressed. First, a limited study population may reduce the results’ external validity, and the results should be interpreted with caution, mainly in regards to the comparison of subgroups in Weber Class A + B and C. In addition, it was not possible to control all the characterization variables, such as the list of drugs, as patients usually use several medications due to their clinical condition. Despite this, we guarantee that patients were undergoing optimized drug treatment. However, this study presents microvascular dynamics during strength exercise for the first time, adding to the understanding of exercise intolerance in HF. We did not perform a reliability NIRS analysis. However, it is noteworthy that the tests were analyzed in duplicate. Additionally, despite considering clinical signs, symptoms, and echocardiographic data to justify the clinical diagnosis, not all patients had their BNP tested; however, the patients were evaluated and diagnosed by cardiologists. Moreover, we did not include a control group. Finally, considering this is the first study evaluating local oxygen extraction during isokinetic muscle strength and echo intensity at rest in HFrEF vs. HFpEF patients, these findings add new insights. Future observations studies with a larger sample size are needed to understand better the effects of the peripheral muscle microcirculation dynamics during strength exercise testing in HF patients.

## 5. Conclusions

Despite similar isokinetic muscle strength and peripheral muscle microcirculatory dynamics parameters during isokinetic muscle strength testing between HF phenotypes, by considering the HF severity, our study reveals a pronounced microcirculatory impairment and slower peripheral recovery following an isokinetic muscle strength testing in HFpEF patients with Class C, coupled with ultrasound-detectable musculoskeletal abnormalities at rest, which are associated with cardiorespiratory capacity.

Moreover, the HFrEF with Weber Class C group participants presented higher values of forced expiratory volume in the first, second, and predicted ratio when comparing them to the HFpEF group. Despite this, no patients needed supplementary oxygen. The groups and subgroups were similar in relation to body mass index.

## Figures and Tables

**Table 1:** Demographic, anthropometric, and clinical characteristics in both heart failure phenotypes.

Parameters	HFpEF		HFrEF		HFpEF	HFrEF	HFpEF vs. HFrEF		Weber Class A + B vs. Weber Class C		HFpEF vs. HFrEF
	Weber Class A + B (*n* = 11)	Weber Class C (*n* = 5)	Weber Class A + B (*n* = 7)	Weber Class C (*n* = 5)	(*n* = 16)	(*n* = 12)	*p*-Value (A + B)	*p*-Value (C)	*p*-Value HFpEF	*p*-Value HFrEF	*p*-Value
	Mean ± SD	Mean ± SD	Mean ± SD	Mean ± SD	Mean ± SD	Mean ± SD					
Male (n, %)	10 (90.9%)	1 (20.0%)	5 (71.4%)	3 (60.0%)	8 (66.7%)	11 (68.8%)	--	--	0.013 ^c,^*	>0.999 ^c^	>0.999 ^c^
Age, years	53.7 ± 9.4	59.8 ± 15.7	53.7 ± 7.9	55.4 ± 7.1	55.6 ± 11.5	54.4 ± 7.3	0.998 ^a^	0.590 ^a^	0.457 ^a^	0.708 ^a^	0.737 ^a^
BMI, kg/m^2^	30.0 ± 3.9	30.1 ± 4.8	28.2 ± 5.5	28.3 ± 5.2	30.0 ± 4.0	28.3 ± 5.1	0.476 ^a^	0.588 ^a^	0.969 ^a^	0.981 ^a^	0.335 ^a^
DXA	(*n* = 11)	(*n* = 4)	(*n* = 4)	(*n* = 3)	(*n* = 15)	(*n* = 7)					
Total body fat mass, %	34.1 ± 3.8	45.4 ± 5.5	36.7 ± 5.8	40.5 ± 10.8	37.1 ± 6.6	38.3 ± 7.8	0.453 ^a^	0.533 ^a^	0.019 ^a,^*	0.624 ^a^	0.724 ^a^
Body fat mass, Kg	29.0 ± 6.9	36.8 ± 6.3	25.5 ± 7.3	31.8 ± 12.0	31.1 ± 7.4	28.2 ± 9.2	0.440 ^a^	>0.999 ^b^	0.087 ^a^	0.400 ^b^	0.582 ^a^
Total body lean mass, %	63.6 ± 3.6	53.0 ± 5.3	61.4 ± 5.6	57.4 ± 9.7	60.8 ± 6.2	59.8 ± 7.2	0.503 ^a^	0.533 ^a^	0.020 ^a,^*	0.567 ^a^	0.732 ^a^
Body lean mass, Kg	55.4 ± 9.0	44.2 ± 6.2	44.7 ± 15.5	46.3 ± 16.4	52.4 ± 9.6	45.4 ± 14.5	0.266 ^a^	0.851 ^a^	0.026 ^a,^*	0.902 ^a^	0.273 ^a^
Right leg fat mass, Kg	3.8 ± 0.8	6.0 ± 1.4	3.2 ± 0.8	4.1 ± 1.9	4.4 ± 1.4	3.6 ± 1.3	0.246 ^a^	0.231 ^a^	0.046 ^a,^*	0.474 ^a^	0.219 ^a^
Right leg lean mass, Kg	9.3 ± 1.9	7.3 ± 1.0	7.1 ± 3.2	6.8 ± 2.8	8.8 ± 1.9	7.0 ± 2.8	0.270 ^a^	0.790 ^a^	0.026 ^a,^*	0.900 ^a^	0.160 ^a^
Left leg fat mass, Kg	3.6 ± 0.7	6.0 ± 1.4	3.2 ± 0.8	4.1 ± 1.8	4.3 ± 1.4	3.6 ± 1.3	0.406 ^a^	0.207 ^a^	0.040 ^a,^*	0.500 ^a^	0.501 ^b^
Left leg lean mass, Kg	9.4 ± 2.0	7.2 ± 0.9	7.0 ± 3.2	6.6 ± 2.4	8.8 ± 2.0	6.8 ± 2.6	0.240 ^a^	0.708 ^a^	0.012 ^a,^*	0.856 ^a^	0.114 ^a^
Heart Diseases											
Ischemic (n, %)	10 (90.9%)	3 (60.0%)	5 (71.4%)	3 (60.0%)	13 (81.3%)	8 (66.7%)	--	--	0.214 ^c^	>0.999 ^c^	0.418 ^c^
Hypertension (n, %)	0 (0.0%)	1 (20.0%)	0 (0.0%)	0 (0.0%)	1 (6.3%)	0 (0.0%)	--	--	0.313 ^c^	>0.999 ^c^	>0.999 ^c^
Idiopathic (n, %)	1 (9.1%)	1 (20.0%)	2 (28.6%)	2 (40.0%)	2 (12.5%)	4 (33.3%)	--	--	>0.999 ^c^	>0.999 ^c^	0.354 ^c^
Risk Factors											
Arterial Hypertension (n, %)	6 (54.6%)	3 (60.0%)	5 (71.4%)	2 (40.0%)	9 (56.3%)	7 (58.3%)	--	--	>0.999 ^c^	0.558 ^c^	>0.999 ^c^
Diabetes Mellitus (n, %)	2 (18.2%)	1 (20.0%)	2 (28.6%)	2 (40.0%)	3 (20.0%)	4 (33.3%)	--	--	>0.999 ^c^	>0.999 ^c^	0.662 ^c^
Dyslipidemia (n, %)	10 (90.9%)	2 (40.0%)	5 (71.4%)	3 (60.0%)	12 (75.0%)	8 (66.7%)	--	--	0.063 ^c^	>0.999 ^c^	0.691 ^c^
Obesity (n, %)	5 (45.5%)	2 (40.0%)	2 (28.6%)	2 (40.0%)	7 (43.8%)	4 (33.3%)	--	--	>0.999 ^c^	>0.999 ^c^	0.705 ^c^
Tabagism (n, %)	2 (18.2%)	3 (60.0%)	4 (57.1%)	2 (40.0%)	5 (31.3%)	6 (50.0%)	--	--	0.245 ^c^	>0.999 ^c^	0.441 ^c^
Coronary Artery Disease (n, %)	8 (72.7%)	2 (40.0%)	2 (28.6%)	3 (60.0%)	10 (62.5%)	8 (66.7%)	--	--	0.300 ^c^	0.558 ^c^	>0.999 ^c^
Drugs											
Beta-blocker (n, %)	10 (90.1%)	5 (100.0%)	7 (100.0%)	5 (100.0%)	15 (93.8%)	12 (100.0%)	--	--	>0.999 ^c^	>0.999 ^c^	>0.999 ^c^
ACEI (n, %)	6 (54.6%)	2 (40.0%)	6 (85.7%)	3 (60.0%)	8 (50.0%)	9 (75.0%)	--	--	>0.999 ^c^	>0.523 ^c^	0.253 ^c^
ARB (n, %)	2 (18.2%)	2 (40.0%)	3 (42.9%)	0 (0.0%)	4 (25.0%)	3 (25.0%)	--	--	>0.547 ^c^	>0.205 ^c^	>0.999 ^c^
Diuretics (n, %)	2 (18.2%)	3 (60.0%)	7 (100.0%)	5 (100.0%)	5 (31.3%)	11 (91.7%)	--	--	>0.245 ^c^	>0.999 ^c^	0.020 ^c,^*
Statins (n, %)	10 (90.9%)	3 (60.0%)	5 (71.4.0%)	3 (60.0%)	13 (81.3%)	8 (66.7%)	--	--	0.214 ^c^	>0.999 ^c^	0.418 ^c^
Coronary Vasodilators (n, %)	1 (9.1%)	2 (40.0%)	1 (14.3%)	1 (20.0%)	3 (18.8%)	2 (16.7%)	--	--	>0.214 ^c^	>0.999 ^c^	>0.999 ^c^
Antidiabetic (n, %)	2 (18.2%)	1 (20.0%)	1 (14.3%)	1 (20.0%)	3 (18.8%)	2 (16.7%)	--	--	>0.999 ^c^	>0.999 ^c^	>0.999 ^c^
Anticoagulants (n, %)	0 (0.0%)	0 (0.0%)	1 (14.3%)	2 (40.0%)	0 (0.0%)	3 (25.0%)	--	--	>0.999 ^c^	0.523 ^c^	0.067 ^c^

Legend: Values are expressed as mean ± standard deviation (SD) or absolute and relative frequencies n (%). Statistics: ^a^ Unpaired t-test; ^b^ Mann–Whitney U test; ^c^ Fisher’s Exact Test. * *p* ≤ 0.05. Abbreviations: HFpEF, heart failure with preserved ejection fraction; HFrEF, heart failure with reduced ejection fraction; BMI, body mass index; kg/m^2^, kilogram per square meter; mL/m^2^, millimeter per square meter; g/m^2^, gram per square meter; DXA, dual-energy X-ray absorptiometry; Kg, kilogram; ACEI, angiotensin-converting enzyme inhibitors; ARB, angiotensin receptor blockers.

**Table 2:** Echocardiogram, pulmonary function, and cardiopulmonary exercise testing parameters in both heart failure phenotypes.

Parameters	HFpEF		HFrEF		HFpEF	HFrEF	HFpEF vs. HFrEF		Weber Class A + B vs. Weber Class C		HFpEF vs. HFrEF
	Weber Class A + B (*n* = 11)	Weber Class C (*n* = 5)	Weber Class A + B (*n* = 7)	Weber Class C (*n* = 5)	(*n* = 16)	(*n* = 12)	*p*-Value (A + B)	*p*-Value (C)	*p*-Value HFpEF	*p*-Value HFrEF	*p*-Value
	Mean ± SD	Mean ± SD	Mean ± SD	Mean ± SD	Mean ± SD	Mean ± SD					
Echocardiogram											
LVEF Simpson (%)	58.4 ± 6.3	59.2 ± 6.3	34.4 ± 4.9	28.6 ± 7.4	58.6 ± 6.1	32.0 ± 6.5	<0.0001 ^b,^*	0.0001 ^a,^*	0.811 ^a^	0.407 ^b^	<0.0001 ^a,^*
LAVI, mL/m^2^	25.0 ± 2.9	29.1 ± 10.4	34.4 ± 5.7	39.4 ± 4.8	26.3 ± 6.2	36.5 ± 5.7	0.004 ^a,^*	0.094 ^a^	0.435 ^a^	0.136 ^a^	<0.0001 ^b,^*
LVMI, g/m^2^	83.8 ± 10.8	95.4 ± 38.4	102.6 ± 33.7	141.0 ± 22.9	87.4 ± 22.4	118.6 ± 34.7	0.197 ^a^	0.059 ^a^	0.543 ^a^	0.041 ^a,^*	0.002 ^b,^*
E/e’, cm/s	6.6 ± 2.1	7.5 ± 0.9	10.9 ± 4.6	11.9 ± 4.7	6.9 ± 1.8	11.3 ± 4.4	0.050 ^a,^*	0.099 ^a^	0.273 ^a^	0.706 ^a^	0.006 ^a,^*
Mean e’ (septal wall), cm/s	8.0 ± 1.9	6.4 ± 1.7	5.1 ± 1.3	3.8 ± 0.8	7.5 ± 2.0	4.6 ± 1.3	0.003 ^b,^*	0.022 ^a,^*	0.127 ^a^	0.096 ^a^	<0.0001 ^a,^*
Mean e’ (lateral wall), cm/s	12.6 ± 3.7	9.4 ± 3.1	8.3 ± 1.9	5.2 ± 1.8	11.6 ± 3.7	7.0 ± 2.3	0.005 ^a,^*	0.040 ^b^*	0.097 ^a^	0.032 ^b,^*	0.001 ^a,^*
Pulmonary Function											
FEV_1_, L/s	2.8 ± 0.8	1.8 ± 0.5	2.7 ± 0.8	2.5 ± 0.6	2.5 ± 0.8	2.6 ± 0.7	0.852 ^a^	0.092 ^a^	0.008 ^a,^*	0.583 ^a^	0.641 ^a^
% Predicted FEV_1_	82.4 ± 23.0	59.6 ± 11.5	82.7 ± 14.7	80.4 ± 12.2	75.3 ± 22.5	81.8 ± 13.2	0.385 ^b^	0.024 ^a,^*	0.035 ^b,^*	0.772 ^a^	0.071 ^b^
Forced Vital Capacity, L	3.8 ± 0.8	2.5 ± 0.6	3.7 ± 1.0	2.9 ± 0.7	3.4 ± 1.0	3.4 ± 0.9	0.774 ^b^	0.277 ^a^	0.003 ^a,^*	0.133 ^b^	0.959 ^a^
% Predicted Forced Vital Capacity	90.4 ± 20.5	67.0 ± 15.3	91.1 ± 10.0	76.0 ± 10.6	83.1 ± 21.6	84.8 ± 12.5	0.339 ^b^	0.316 ^a^	0.064 ^b^	0.036 ^a,^*	0.788 ^a^
FEV_1_/FVC, %	72.8 ± 6.6	72.6 ± 7.4	73.0 ± 7.9	85.4 ± 6.3	72.8 ± 6.6	78.2 ± 9.5	0.808 ^b^	0.020 ^a,^*	0.957 ^a^	0.005 ^b,^*	0.106 ^a^
% Predicted FEV_1_/FVC	90.2 ± 6.7	89.2 ± 6.6	87.2 ± 8.1	92.0 ± 17.8	89.9 ± 6.5	89.2 ± 12.5	0.426 ^a^	>0.999 ^b^	0.792 ^a^	0.965 ^b^	0.442 ^b^
Cardiopulmonary exercise testing											
Exercise, min	10.8 ± 2.5	6.6 ± 0.9	10.1 ± 2.6	6.9 ± 2.2	9.5 ± 2.9	8.8 ± 2.9	0.641 ^b^	0.782 ^a^	0.001 ^b,^*	0.048 ^a,^*	0.639 ^b^
Peak RER	1.2 ± 0.1	1.3 ± 0.1	1.2 ± 0.1	1.3 ± 0.1	1.3 ± 0.1	1.3 ± 0.1	0.643 ^b^	>0.999 ^a^	0.179 ^b^	0.191 ^a^	0.859 ^b^
Peak Power Output, W	140.3 ± 27.0	84.8 ± 15.3	107.3 ± 26.0	74.8 ± 21.7	122.9 ± 35.9	93.8 ± 28.6	0.024 ^a,^*	0.426 ^a^	0.0002 ^a,^*	0.041 ^a,^*	0.024 ^a,^*
Peak HR, bpm	141.9 ± 18.0	116.8 ± 28.3	130.6 ± 16.5	125.2 ± 25.6	133.8 ± 24.2	128.3 ± 19.9	0.192 ^a^	0.605 ^a^	0.113 ^a^	0.694 ^a^	0.516 ^a^
Peak VO_2_, mL·kg^−1^·min^−1^	22.1 ± 3.4	14.4 ± 1.2	19.2 ± 2.7	13.7 ± 1.6	19.7 ± 4.7	16.9 ± 3.6	0.060 ^a^	0.434 ^a^	<0.0001 ^a,^*	0.001 ^a,^*	0.081 ^a^
% Predicted peak VO_2_, mL·kg^−1^.min^−1^	66.0 ± 9.1	54.3 ± 17.1	58.8 ± 10.7	43.4 ± 7.5	62.3 ± 12.8	52.4 ± 12.1	0.167 ^a^	0.151 ^b^	0.052 ^b^	0.048 ^b,^*	0.046 ^a,^*
Peak VO_2_, mL·min^−1^	1884.6 ± 312.7	1180.6 ± 126.2	1469.6 ± 344.0	1034.8 ± 251.5	1664.6 ± 427.8	1288.4 ± 371.0	0.024 ^a,^*	0.291 ^a^	<0.0001 ^a,^*	0.030 ^a,^*	0.020 ^a,^*
VE/VCO_2_ Slope, L/min	27.9 ± 3.7	28.8 ± 8.0	31.1 ± 5.4	30.1 ± 4.0	28.2 ± 5.1	30.7 ± 4.6	0.196 ^a^	0.310 ^b^	0.510 ^b^	0.876 ^b^	0.084 ^b^

Legend: Values are expressed as mean ± standard deviation (SD) or absolute and relative frequencies *n* (%). Statistics: ^a^ Unpaired *t*-test; ^b^ Mann–Whitney U test. * *p* ≤ 0.05. Abbreviations: HFpEF, heart failure with preserved ejection fraction; HFrEF, heart failure with reduced ejection fraction; LVEF, left ventricular ejection fraction; LAVI, left atrial volume index; LVMI, left ventricular mass index E/e’, early mitral inflow velocity and mitral annular early diastolic velocity ratio; cm/s, centimeters per second; FEV_1_, forced expiratory volume in the first second; L/s, liters per second; L, liter; FEV_1_/CVF, the proportion of vital capacity that they are able to expire in the first second of forced expiration to the full, forced vital capacity; min, minute; W, watt; RER, respiratory gas exchange ratio; HR, heart rate; VO_2_, oxygen uptake; mL·kg^−1^·min^−1^, millimeter per minute per kilogram; ml/min, millimeter per minute; VE/VCO_2_, minute ventilation/carbon dioxide production slope; L/min, liters per minute.

**Table 3:** NIRS during isokinetic muscle strength parameters in both heart failure phenotypes and Weber Class.

Parameters	HFpEF		HFrEF		HFpEF	HFrEF	HFpEF vs. HFrEF		Weber Class A + B vs. Weber Class C		HFpEF vs. HFrEF
	Weber Class A + B (*n* = 6)	Weber Class C (*n* = 4)	Weber Class A + B (*n* = 3)	Weber Class C (*n* = 4)	(*n* = 10)	(*n* = 7)	*p*-Value	*p*-Value	*p*-Value	*p*-Value	*p*-Value
	Mean ± SD (95% CI)	Mean ± SD (95% CI)	Mean ± SD (95% CI)	Mean ± SD (95% CI)	Mean ± SD (95% CI)	Mean ± SD (95% CI)	Mean Difference A + B (95% CI)	Mean Difference C (95% CI)	Mean Difference HFpEF (95% CI)	Mean Difference HFrEF (95% CI)	Mean Difference (95% CI)
TSI (%)											
Baseline	73.7 ± 2.2 (71.3 to 76.0)	72.0 ± 5.1 (63.9 to 80.2)	73.4 ± 6.1 (58.3 to 88.6)	70.5 ± 2.0 (67.3 to 73.7)	73.0 ± 3.5 (70.5 to 75.5)	71.8 ± 4.1 (68.0 to 75.6)	0.958 ^a^ 0.21 (−13.8 to 14.2)	0.616 ^a^ 1.5 (−6.2 to 9.2)	0.578 ^a^ −1.7 (−9.4 to 6.1)	0.497 ^a^ −2.9 (16.7 to 10.9)	0.532 ^a^ 1.2 (−2.9 to 5.4)
Exercise	44.2 ± 8.2 (35.7 to 52.8)	57.6 ± 13.7 (35.9 to 79.4)	36.0 ± 2.4 (30.1 to 41.9)	44.8 ± 6.6 (34.3 to 55.3)	49.6 ± 12.1 (40.9 to 58.3)	41.0 ± 6.7 (34.8 to 47.2)	0.060 ^a^ 8.2 (−0.5 to 16.9)	0.161 ^a^ 12.9 (−7.6 to 33.3)	0.146 ^a^ 13.4 (−6.9 to 33.7)	0.071 ^a^ 8.8 (−1.2 to 18.8)	0.082 ^a^ 8.6 (−1.3 to 18.5)
Recovery	64.1 ± 8.5 (55.1 to 73.0)	65.1 ± 13.5 (43.6 to 86.5)	58.1 ± 15.3 (20.2 to 96.1)	73.8 ± 3.7 (67.9 to 79.7)	64.5 ± 10.1 (57.3 to 71.7)	67.1 ± 12.5 (55.6 to 78.6)	0.583 ^a^ 5.9 (−26.7 to 38.5)	0.290 ^a^ −8.7 (−29.4 to 12.0)	0.899 ^a^ 1.0 (−19.0 to 21.0)	0.214 ^a^ 15.7 (−20.3 to 51.6)	0.475 ^b^ −2.6 (−14.7 to 8.4)
O_2_Hb (μM)											
Baseline	0.0 ± 1.2 (−1.3 to 1.3)	0.0 ± 0.6 (−0.9 to 0.9)	−0.2 ± 0.3 (−1.3 to 1.3)	0.7 ± 0.9 (−0.5 to 1.5)	0.0 ± 1.0 (−0.7 to 0.7)	0.3 ± 0.8 (−0.4 to 1.0)	0.712 ^a^ −0.2 (−1.1 to 1.5)	0.245 ^a^ −0.7 (−2.0 to 0.6)	0.990 ^a^ 0.0 (−1.4 to 1.3)	0.132 ^a^ 0.9 (−0.4 to 2.2)	0.475 ^b^ −0.2 (−1.4 to 0.5)
Exercise	−27.2 ± 9.2 (−36.8 to −17.6)	−10.9 ± 3.8 (−17.0 to −4.8)	−30.0 ± 6.7 (−46.5 to −13.4)	−23.7 ± 5.7 (−32.8 to −14.5)	−20.7 ± 11.1 (−28.7 to −12.8)	−26.4 ± 6.5 (−32.4 to −20.3)	0.626 ^a^ 2.8 (−10.6 to 16.2)	0.029 ^b,^* 13.5 (0.1 to 21.2)	0.006 ^a,^* 16.3 (6.4 to 26.2)	0.400 ^b^ 5.8 (−2.6 to 22.4)	0.204 ^a^ 5.7 (−3.4 to 14.8)
Recovery	−4.1 ± 7.9 (−12.4 to 4.2)	−3.0 ± 3.4 (−8.4 to 2.4)	−4.5 ± 8.3 (−25.1 to 16.2)	5.9 ± 2.8 (1.4 to 10.4)	−3.7 ± 6.2 (−8.2 to 0.8)	1.5 ± 7.6 (−5.6 to 8.5)	0.957 ^a^ 0.3 (−15.9 to 16.5)	0.007 ^a,^* −8.9 (−14.4 to −3.5)	0.766 ^a^ 1.1 (−7.4 to 9.7)	0.154 ^a^ 10.4 (−8.3 to 29.1)	0.167 ^a^ −5.2 (−12.8 to 2.5)
HHb (μM)											
Baseline	−0.1 ± 0.3 (−0.4 to 0.2)	−0.2 ± 0.4 (−0.7 to 0.4)	−0.1 ± 0.2 (−0.6 to 0.5)	0.5 ± 0.6 (−0.5 to 1.0)	−0.1 ± 0.3 (−0.3 to 0.1)	0.2 ± 0.5 (−0.2 to 0.7)	0.809 ^a^ −0.04 (−0.5 to 0.4)	0.128 ^a^ −0.6 (−1.5 to 0.3)	0.866 ^a^ 0.0 (−0.6 to 0.5)	0.160 ^a^ 0.5 (−0.3 to 1.4)	0.124 ^a^ −0.4 (−0.9 to 0.1)
Exercise	14.0 ± 6.4 (7.4 to 20.7)	3.4 ± 7.6 (−8.7 to 15.5)	11.6 ± 5.2 (−1.2 to 24.5)	8.3 ± 4.3 (1.4 to 15.2)	10.7 ± 8.2 (4.8 to 16.5)	13.1 ± 6.5 (7.1 to 19.1)	0.571 ^a^ 2.4 (−7.7 to 12.5)	0.313 ^a^ −4.9 (−16.4 to 6.5)	0.062 ^a^ −10.7 (−22.1 to 0.7)	0.415 ^a^ −3.4 (−13.6 to 6.9)	0.504 ^a^ −2.4 (−10.1 to 5.2)
Recovery	8.9 ± 5.6 (3.0 to 14.8)	4.6 ± 6.1 (−5.0 to 14.4)	18.8 ± 4.8 (6.9 to 30.8)	0.7 ± 1.7 (−2.0 to 3.5)	6.9 ± 6.1 (2.5 to 11.3)	5.4 ± 6.7 (−0.8 to 11.6)	0.042 ^a,^* −10.0 (−19.4 to −0.5)	0.289 ^a^ 4.0 (−5.4 to 13.3)	0.313 ^a^ −4.2 (−13.5 to 5.1)	0.016 ^a,^* −18.1 (−28.9 to −7.3)	0.652 ^a^ −1.5 (−5.4 to 8.4)

Legend: Values are expressed as mean ± standard deviation (SD), mean difference (MD), and 95% confidence interval (95% CI) or median difference, number of included patients (n). Statistics: ^a^ Unpaired *t*-test (MD and 95% CI); ^b^ Mann–Whitney test (Hodge–Lehmann’s median difference considered); * *p* ≤ 0.05. Abbreviations: HFpEF, heart failure with preserved ejection fraction; HFrEF, heart failure with reduced ejection fraction; TSI, tissue saturation index; O_2_Hb, oxyhemoglobin; HHb, deoxyhemoglobin.

**Table 4:** Isokinetic muscle strength parameters in both heart failure phenotypes and Weber Class.

Parameters	HFpEF		HFrEF		HFpEF	HFrEF	HFpEF vs. HFrEF		Weber Class A + B vs. Weber Class C		HFpEF vs. HFrEF
	Weber Class A + B (*n* = 6)	Weber Class C (*n* = 4)	Weber Class A + B (*n* = 3)	Weber Class C (*n* = 4)	(*n* = 10)	(*n* = 7)	*p*-Value	*p*-Value	*p*-Value	*p*-Value	*p*-Value
	Mean ± SD (95% CI)	Mean ± SD (95% CI)	Mean ± SD (95% CI)	Mean ± SD (95% CI)	Mean ± SD (95% CI)	Mean ± SD (95% CI)	Mean Difference A + B (95% CI)	Mean Difference C (95% CI)	Mean Difference HFpEF (95% CI)	Mean Difference HFrEF (95% CI)	Mean Difference (95% CI)
PT, Nm	125.5 ± 25.7 (98.6 to 152.4)	68.2 ± 10.8 (51.1 to 85.3)	136.8 ± 47.5 (18.7 to 254.8)	83.4 ± 38.8 (21.6 to 145.1)	102.6 ± 35.8 (77.0 to 128.2)	106.3 ± 48.2 (61.7 to 150.8)	>0.999 ^b^ −2.0 (−74.4 to 47.6)	0.500 ^a^ −15.2 (−74.7 to 44.4)	0.019 ^b,^* 64.8 (11.4 to 83.6)	0.190 ^a^ 53.4 (−41.3 to 148.1)	0.887 ^b^ 10.4 (−49.8 to 37.2)
PT/Body Mass, Nm.kg	139.2 ± 28.9 (108.9 to 169.6)	82.4 ± 1.9 (79.4 to 85.3)	159.7 ± 32.6 (78.6 to 240.7)	105.2 ± 37.9 (45.0 to 165.5)	116.5 ± 36.4 (90.4 to 142.5)	128.6 ± 43.8 (88.0 to 169.1)	0.414 ^a^ −20.5 (−84.6 to 43.7)	0.314 ^a^ −22.9 (−83.0 to 37.3)	0.005*^a^ 56.8 (26.5 to 87.2)	0.099 ^a^ 54.4 (−15.1 to 123.9)	0.561 ^a^ −12.1 (−56.3 to 32.1)
Maximal Repetition Total Work, J	143.5 ± 32.2 (109.7 to 177.3)	79.5 ± 15.7 (54.4 to 104.5)	151.5 ± 43.9 (42.6 to 260.5)	99.4 ± 45.2 (27.5 to 171.4)	117.9 ± 41.9 (87.9 to 147.8)	121.8 ± 49.4 (76.1 to 167.4)	0.796 ^a^ −8.0 (−96.7 to 80.7)	0.454 ^a^ −20.0 (−88.5 to 48.6)	0.003 ^a,^* 64.1 (28.4 to 99.7)	0.191 ^a^ 52.1 (−37.9 to 142.1)	0.869 ^a^ −3.9 (−53.9 to 46.2)
Work/Body Weight, %	159.2 ± 36.6 (120.8 to 197.7)	95.9 ± 10.5 (79.1 to 112.6)	177.9 ± 26.3 (112.5 to 243.3)	125.1 ± 40.9 (60.0 to 190.1)	133.9 ± 43.1 (103.1 to 164.7)	147.7 ± 43.2 (107.8 to 187.6)	0.417 ^a^ −18.7 (−71.7 to 34.3)	0.251 ^a^ −29.2 (−92.1 to 33.8)	0.007 ^a,^* 63.4 (24.8 to 101.9)	0.093 ^a^ 52.9 (−12.8 to 118.5)	0.527 ^a^ −13.8 (−59.7 to 32.1)
Total Work, J	2253.5 ± 523.0 (1704.6 to 2802.3)	1245.4 ± 193.5 (937.5 to 1553.2)	2351.1 ± 674.5 (675.7 to 4026.6)	1398.2 ± 593.7 (453.5 to 2342.9)	1850.2 ± 659.9 (1378.2 to 2322.3)	1806.6 ± 766.4 (1097.9 to 2515.4)	0.839 ^a^ −98.7 (−1448.2 to 1252.8)	0.653 ^a^ −152.9 (−1055.7 to 749.9)	0.004 ^a,^* 1008.1 (450.3 to 1565.9)	0.122 ^a^ 952.9 (−396.0 to 2301.8)	0.905 ^a^ 43.6 (−736.2 to 823.4)
Work Fatigue, %	36.5 ± 11.0 (24.9 to 48.0)	32.8 ± 19.2 (2.2 to 63.4)	40.0 ± 9.3 (17.0 to 63.0)	48.1 ± 2.0 (44.9 to 51.3)	35.0 ± 13.9 (25.0 to 45.0)	44.6 ± 7.0 (38.1 to 51.1)	0.634 ^a^ −3.6 (−21.7 to 14.6)	0.209 ^a^ −15.3 (−45.7 to 15.1)	0.747 ^a^ 3.7 (−24.9 to 32.2)	0.267 ^a^ −8.1 (−30.1 to 13.9)	0.133 ^b^ −8.3 (−15.1 to 1.7)
Average Power, W	189.4 ± 47.1 (140.0 to 238.8)	103.0 ± 22.7 (66.9 to 139.1)	206.4 ± 78.1 (12.4 to 400.3)	114.4 ± 54.6 (27.6 to 201.2)	154.8 ± 58.2 (113.1 to 196.5)	153.8 ± 77.1 (82.6 to 225.1)	0.753 ^a^ −17.0 (−181.1 to 147.1)	0.719 ^a^ −11.4 (−93.4 to 70.6)	0.005 ^a,^* 86.4 (34.4 to 138.4)	0.168 ^a^ 92.0 (−64.6 to 248.4)	0.978 ^a^ 1.0 (−75.2 to 77.2)

Legend: Values are expressed as mean ± standard deviation (SD), mean difference (MD), and 95% confidence interval (95% CI) or median difference, number of included patients (n). Statistics: ^a^ Unpaired *t*-test (MD and 95% CI); ^b^ Mann–Whitney test (Hodge–Lehmann’s median difference considered); * *p* ≤ 0.05. Abbreviations: HFpEF, heart failure with preserved ejection fraction; HFrEF, heart failure with reduced ejection fraction; PT, peak torque to Nm, newton-meter; J, Joules; W, Watt.

**Table 5:** Ultrasound-derived parameters (echo intensity and muscle thickness) in both heart failure phenotypes and Weber Class.

Parameters	HFpEF		HFrEF		HFpEF	HFrEF	HFpEF vs. HFrEF		Weber Class A + B vs. Weber Class C		HFpEF vs. HFrEF
	Weber Class A + B (*n* = 11)	Weber Class C (*n* = 5)	Weber Class A + B (*n* = 7)	Weber Class C (*n* = 5)	(*n* = 16)	(*n* = 12)	*p*-Value	*p*-Value	*p*-Value	*p*-Value	*p*-Value
	Mean ± SD (95% CI)	Mean ± SD (95% CI)	Mean ± SD (95% CI)	Mean ± SD (95% CI)	Mean ± SD (95% CI)	Mean ± SD (95% CI)	Mean Difference A + B (95% CI)	Mean Difference C (95% CI)	Mean Difference HFpEF (95% CI)	Mean Difference HFrEF (95% CI)	Mean Difference (95% CI)
Echo intensity, 0–255											
m. rectus femoris	14.1 ± 8.7 (8.2 to 19.9)	29.7 ± 8.4 (19.3 to 40.2)	13.1 ± 5.5 (8.0 to 18.2)	15.1 ± 6.8 (6.8 to 23.5)	19.0 ± 11.2 (13.0 to 25.0)	13.9 ± 5.9 (10.2 to 17.7)	0.786 ^a^ 0.9 (−6.2 to 8.1)	0.017 ^a,^* 14.6 (3.4 to 25.9)	0.009 ^a,^* −15.7 (−26.2 to −5.2)	0.606 ^a^ −2.0 (−10.6 to 6.6)	0.140 ^a^ 5.0 (−1.8 to 11.8)
Muscle thickness, cm											
m. rectus femoris	2.1 ± 0.5 (1.7 to 2.4)	1.7 ± 0.3 (1.3 to 2.1)	1.9 ± 0.4 (1.5 to 2.3)	1.5 ± 0.5 (0.9 to 2.1)	2.0 ± 0.5 (1.7 to 2.2)	1.7 ± 0.5 (1.4 to 2.0)	0.435 ^b^ 0.2 (−0.3 to 0.9)	0.397 ^a^ 0.2 (−0.4 to 0.8)	0.134 ^a^ 0.4 (−0.1 to 0.8)	0.145 ^b^ 0.3 (−0.2 to 1.0)	0.196 ^a^ 0.2 (−0.1 to 0.6)
	(*n* = 10)	(*n* = 4)	(*n* = 7)	(*n* = 5)							
m. quadriceps femoris	3.8 ± 0.7 (3.3 to 4.3)	3.0 ± 0.4 (2.3 to 3.6)	3.6 ± 0.8 (2.8 to 4.3)	2.8 ± 1.0 (1.6 to 4.0)	3.6 ± 0.7 (3.1 to 4.0)	3.2 ± 0.9 (2.6 to 3.8)	0.570 ^a^ 0.2 (−0.6 to 1.1)	0.734 ^a^ 0.2 (−1.1 to 1.4)	0.023 ^a,^* 0.8 (0.1 to 1.5)	0.203 ^a^ 0.8 (−1.5 to 2.0)	0.364 ^a^ 0.4 (−0.6 to 1.3)

Legend: Values are expressed as mean ± standard deviation (SD), mean difference (MD) and 95% confidence interval (95% CI) or median difference, number of included patients (n). Statistics: ^a^ Unpaired *t*-test (MD and 95% CI); ^b^ Mann–Whitney test (Hodge–Lehmann’s median difference considered); * *p* ≤ 0.05. Abbreviations: HFpEF, heart failure with preserved ejection fraction; HFrEF, heart failure with reduced ejection fraction; echo intensity, 0 = black, and 255 = white; m, muscle; cm, centimeters.

**Table 6:** Associations between tissue saturation index response via near-infrared spectroscopy, rectus femoris echo intensity, and muscle thickness with isokinetic muscle strength and cardiorespiratory fitness in both HF phenotypes (HFpEF and HFrEF) and according to disease severity (Weber A + B and Weber C).

Groups		TSI × PT	TSI × peak VO_2_	RF_EI × PT	RF_EI × peak VO_2_	RF_MT × PT	RF_MT × Peak VO_2_
HFpEF (Weber A + B)	r	−0.429 ^b^	0.008 ^a^	−0.331 ^a^	−0.060 ^a^	0.235 ^a^	0.687 ^a^
	p	0.419	0.988	0.320	0.861	0.487	0.020 *
HFrEF (Weber A + B)	r	0.626 ^a^	0.999 ^a^	0.151 ^a^	0.294 ^a^	0.649 ^a^	0.612 ^a^
	p	0.569	0.010 *	0.747	0.522	0.114	0.144
HFpEF (Weber C)	r	−0.417 ^a^	−0.786 ^a^	0.121 ^a^	−0.537 ^a^	0.405 ^a^	0.937 ^a^
	p	0.583	0.214	0.847	0.350	0.499	0.019 *
HFrEF (Weber C)	r	0.311 ^a^	0.476 ^a^	−0.228 ^a^	0.152 ^a^	0.880 ^a^	0.838 ^a^
	p	0.689	0.524	0.713	0.807	0.049 *	0.077
HFpEF	r	−0.697 ^b^	−0.586 ^a^	−0.570 ^a^	−0.581 ^a^	0.398 ^a^	0.672 ^a^
	p	0.031 *	0.075	0.021 *	0.018*	0.127	0.004 *
HFrEF	r	−0.229 ^a^	−0.291 ^a^	−0.074 ^a^	0.079 ^a^	0.778 ^a^	0.751 ^a^
	p	0.621	0.527	0.820	0.808	0.003*	0.005 *

Legend: Values are expressed as absolute values. Statistics: ^a^ Person’s correlation test (r correlation coefficient with 0.00 no association; 0.20 weakly; 0.50 moderately; 0.8 strongly and 1.00 perfectly) and ^b^ Spearman’s correlation test (rho correlation coefficient with 0.00 to 0.20 negligible; 021 to 0.40 weak; 0.41 to 0.60 moderate; 0.61 to 0.80 strong and 0.81 to 1.00 very strong); * *p* ≤ 0.05, correlation coefficient of 0.40–0.59 were considered moderate. Abbreviations: HFpEF, heart failure with preserved ejection fraction; HFrEF, heart failure with reduced ejection fraction; TSI, tissue saturation index (%); PT, peak torque (Nm); peak VO_2_, oxygen uptake (mL·min^−1^); RF_EI, rectus femoris echo intensity; RF_MT, rectus femoris muscle thickness.

## Data Availability

Data available on request due to restrictions of a private or ethical nature. The data presented in this study are available on request from the corresponding author. The data are not publicly available due to reasons of sensitivity e.g., human data, patient localization, etc.
